# Room-temperature Pd/Ag direct arylation enabled by a radical pathway

**DOI:** 10.3762/bjoc.16.36

**Published:** 2020-03-13

**Authors:** Amy L Mayhugh, Christine K Luscombe

**Affiliations:** 1 Department of Chemistry, University of Washington, Seattle, WA 98195, USA; 2 Department of Materials Science & Engineering, University of Washington, Seattle, WA 98195, USA

**Keywords:** direct arylation, indole, palladium radical, visible light

## Abstract

Direct arylation is an appealing method for preparing π-conjugated materials, avoiding the prefunctionalization required for traditional cross-coupling methods. A major effort in organic electronic materials development is improving the environmental and economic impact of production; direct arylation polymerization (DArP) is an effective method to achieve these goals. Room-temperature polymerization would further improve the cost and energy efficiencies required to prepare these materials. Reported herein is new mechanistic work studying the underlying mechanism of room temperature direct arylation between iodobenzene and indole. Results indicate that room-temperature, Pd/Ag-catalyzed direct arylation systems are radical-mediated. This is in contrast to the commonly proposed two-electron mechanisms for direct arylation and appears to extend to other substrates such as benzo[*b*]thiophene and pentafluorobenzene.

## Introduction

π-Conjugated polymers are of significant interest as they have the potential to combine the mechanical flexibility and affordability of synthetic polymers with the optical and electronic properties of semiconductors. One of the limitations to this field’s continued growth are the expensive, toxic, and energy-intensive methods in which these materials are typically prepared [1]. Direct arylation is one solution to these problems, which allows improved atom and step economy in polymer synthesis. Traditional coupling methods form a new C–C bond using a reactive C–M bond (M = SnR_3_, B(OH)_2_, etc.) and a C–X (X = I, Br, OTs) coupling partner. In contrast, direct arylation couples C–H and C–X bonds directly without the need to prepare the C–M substrate. As a polymerization method, direct arylation polymerization (DArP) is of great interest due to its potential to reduce waste and toxicity by removing the need for stoichiometric amounts of toxic organometallics [2].

While significant progress has been made in DArP, C–H bonds are more challenging to functionalize than their C–M counterparts. Known DArP conditions have limited monomers that can produce polymers with both high molecular weight and regioregularity, and elevated reaction temperatures are typically required [3]. Mild conditions for C–H functionalization is of growing interest within the broader synthetic community as an opportunity to improve functional group tolerance, and environmental impact [4–5]. While many direct arylation systems occur under neutral conditions and without the need for strong oxidants or reductants, only few proceed at ambient temperatures [3,6]. To our knowledge, only one example of room-temperature DArP exists, namely polymerizing poly-3-hexylthiophene to an acceptably high number average molecular weight of 14 kg/mol, although in 9% yield [7]. Room-temperature DArP would improve the simplicity of conjugated polymer synthesis, and reduce the energy requirements.

**Scheme 1:**
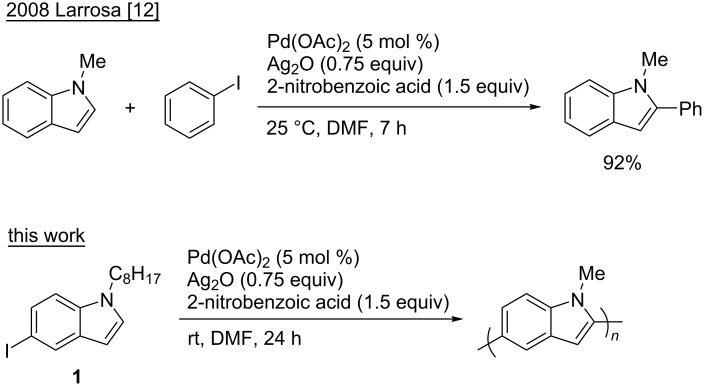
A high yielding, highly selective room-temperature direct arylation reaction between indole and iodobenzene reported in ref. [12] and adapted in this work to study room-temperature DArP.

Small molecule reactions can help predict a method’s utility in polymerizations, as DArP proceeds through a step-growth polymerization mechanism. Generically, a small molecule reaction is a candidate for adaptation to a polymerization when it is highly regioselective, and high yielding. Under step-growth conditions a high yielding reaction is essential for producing a high molecular weight material [8], which directly influences the quality and electronic performance of the material [7,9]. More specific to this work, the direct arylation method should be catalytic, directing-group free and at room temperature; a handful of such methods have been reported [10–14]. One such method, an indole/iodoarene direct arylation method reported by Larrosa is notable: proceeding in high yields, at room temperature, and with no reported regioselectivity issues (Scheme 1) [12]. This system is hypothesized to proceed under mild conditions due to a highly electrophilic Pd catalyst generated in situ. In this paper, we investigated the mechanism of room-temperature direct arylation and DArP by extending the method reported by Larrosa to synthesize a conjugated polymer, polyindole (PIn). Small molecule mechanistic studies were undertaken that will help future development of mild, efficient DArP conditions.

## Results and Discussion

**Figure 1:**
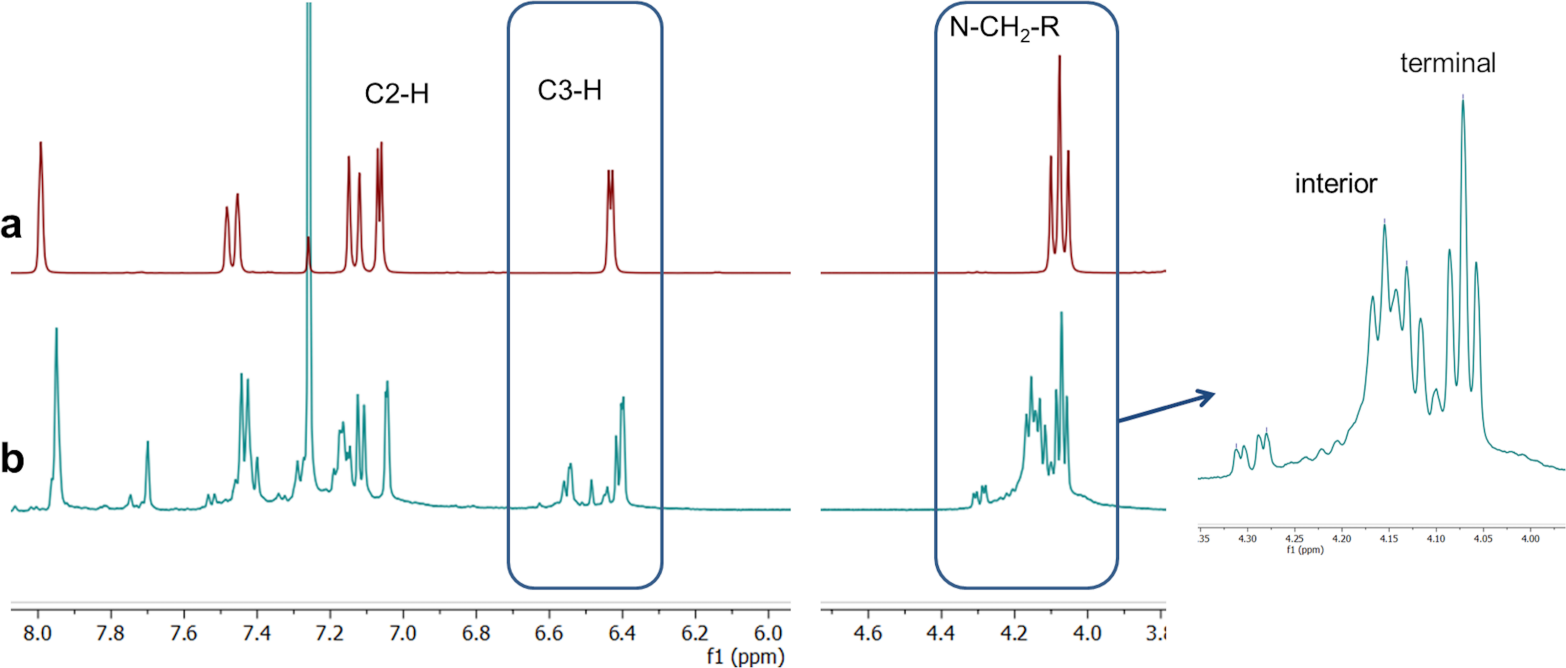
^1^H NMR (500 MHz, CDCl_3_) of (a) 5-iodo-1-octylindole monomer (b) PIn prepared according to conditions in Scheme 1. The region from 4–4.5 ppm indicates the interior indole repeat units compared with the terminal indole units.

**Figure 2:**
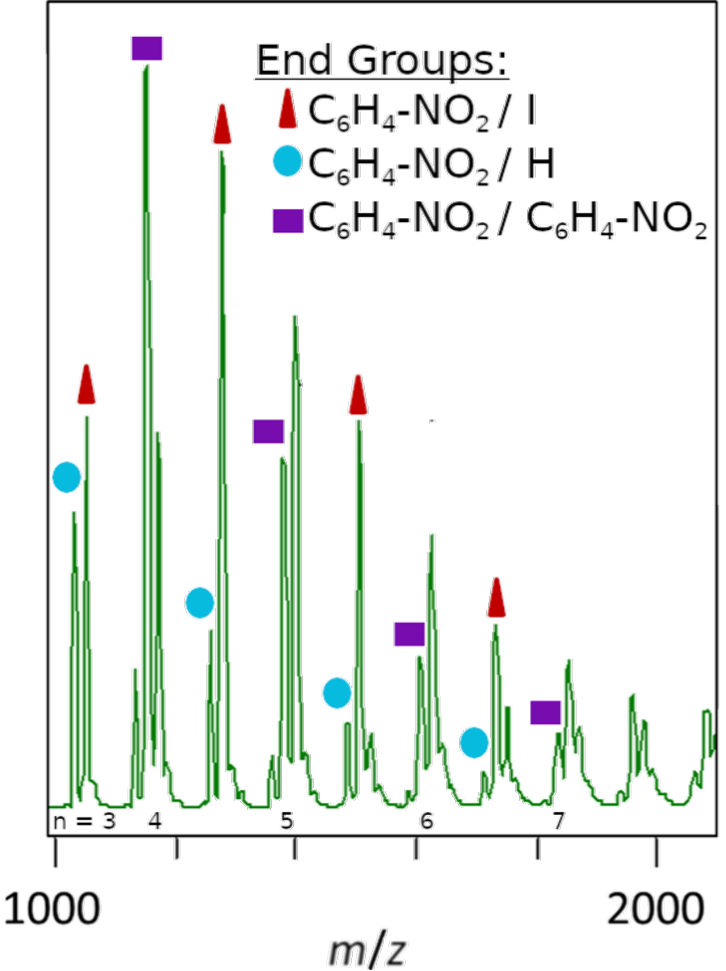
MALDI–TOF MS of PIn, indicating octylindole repeat units with three different types of end groups. These include 2-nitrophenyl, iodine, and hydrogen.

Initial attempts to polymerize iodoindole monomer **1** using the reported conditions (Scheme 1) produced oligomers with an unanticipated coupling pattern in good yield (87%, Figure 1, Supporting Information File 1, Figure S3). The ^1^H NMR signals from the C2-H, C3-H on the pyrrole ring and the N-CH_2_-R on the alkyl chain indicate the product is highly branched [15]. Comparing the monomer’s spectrum to the polymer’s shows large peaks from unfunctionalized pyrrole rings at the chain ends with a ratio of interior to end groups of 1:0.57. While low molecular weight also contributes to the large signals from the chain ends, there are a few factors that indicate branching is occurring: firstly, the large variety in C3-H signals; secondly, the relative integration of the C2-H/C3-H signals to the N-CH_2_-R signals show that for every indole unit, there is less than one H on the pyrrole ring. Finally, branching is evidenced by the discrepancy in molecular weight as calculated for a linear polymer by ^1^H NMR compared with that indicated by MALDI–TOF MS (Figure 2). Due to the high regioselectivity in small molecule couplings, defects to this extent were unexpected.

MALDI–TOF MS was collected for PIn to further clarify the nature of the coupling pattern (Figure 2, Supporting Information File 1, Figure S2). The repeat unit corresponds to C/H–C/I coupling (*m*/*z* = 227) as expected for direct arylation. In contrast to the expected I/H end groups, the mass spectrum indicates various incorporations of nitrobenzene into the polymer chains. The 2-nitrobenzoic acid used to form a silver carboxylate in the reaction system is the origin of the nitrophenyl group. Three different types of chains are represented, corresponding to incorporation of I/nitrophenyl, H/nitrophenyl, and two nitrophenyl end groups.

2-Substituted benzoic acids in general, and 2-nitrobenzoic acid in particular, are reactive substrates in decarboxylative coupling reactions [16–17]. However, more forceful conditions are typically required than used in the room-temperature system. Indeed, a very similar Pd/Ag system has been reported for decarboxylative coupling between indole and 2-nitrobenzoic acids at 110 °C [18]. Under such conditions, silver carboxylates decompose to produce carbonyl and phenyl radicals, which could explain the origin of nitrobenzene incorporation [19–20]. When the radical trapping agent BHT was added to the polymerization conditions, no polymerization occurred (Table 1). Additionally, the reaction was completely inhibited when run in the dark. These results indicate that visible light may be responsible for radical generation in the polymerization.

**Table 1:** Polymerization control experiments.

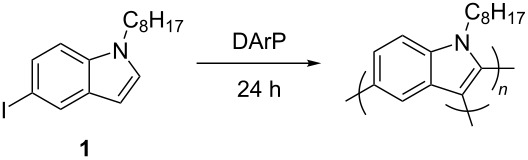		

	Variation	Yield (%)

1	+ BHT^a,b^	0
2	dark	0
3	no Pd(OAc)_2_	0

^a^(2,6-Di-*tert*-butyl-4-methylphenol); ^b^1 equiv was used; conditions: Pd(OAc)_2_ (5 mol %), Ag_2_O (0.75 equiv), 2-nitrobenzoic acid (1.5 equiv), DMF, rt.

To further investigate the possibility of a radical-mediated reaction, additional radical capture experiments were performed for the small molecule reaction using 1-methylindole and iodobenzene (Table 2). Similar results were observed for 1-octylindole and iodobenzene (Supporting Information File 1, Table S2). In all cases, coupling was completely suppressed by the addition of TEMPO and BHT. Moreover, Ph-BHT was observed by GC–MS when BHT was added. Similar to the polymerization trials, coupling was inhibited when the standard conditions were run in the dark. Both the Ph-BHT adduct and continued poor reactivity in the dark provides growing evidence for a radical mechanism. Moreover, the observed behavior appears to be common between the iodoindole monomer and iodobenzene/indole system, rather than a phenomenon unique to an electron-rich iodoindole.

**Table 2:** Small molecule control experiments.

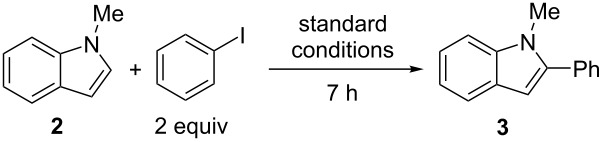		

	Variation	Yield (%)^a^

1	none	85^b^
2	+ BHT^c,d^	0
3	+ TEMPO^c,e^	0
4	dark	20

^a^Determined by ^1^H NMR using ethylene carbonate as an internal standard; ^b^isolated yield; ^c^2 equiv was used; ^d^Ph-BHT adduct observed by GC–MS; ^e^((2,2,6,6-tetramethylpiperidin-1-yl)oxyl); conditions: Pd(OAc)_2_ (5 mol %), Ag_2_O (0.75 equiv), 2-nitrobenzoic acid (1.5 equiv), DMF, rt.

**Scheme 2:**
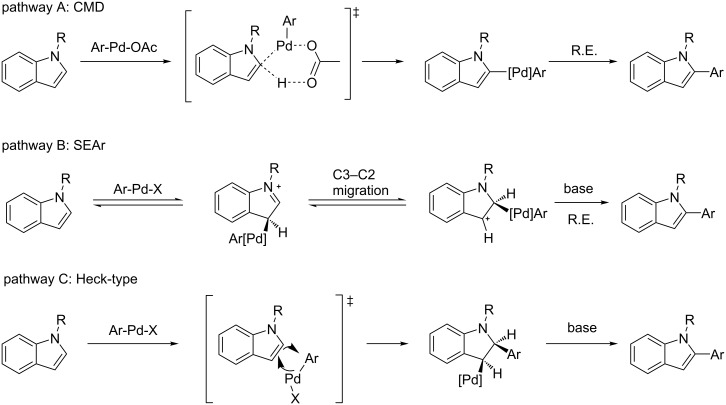
Commonly discussed mechanisms for C2 selective direct arylation, none containing radical intermediates.

The growing evidence for a phenyl radical in the catalytic cycle is in contrast to the typically proposed mechanisms for direct arylation (Scheme 2). Amongst these mechanisms, the most widely accepted is the concerted metalation–deprotonation (CMD) pathway [21]. Within the indole direct-arylation literature, however, there remains much discussion of an electrophilic metalation mechanism, with the majority of experimental evidence supporting this pathway [22–26]. In this case, C2 selectivity is often observed, theoretically from a C3/C2 Pd migration. A third possible mechanism that has had little experimental evidence but cannot be ruled out, is a Heck-type carbopalladation. This mechanism has been recently supported by ^13^C and ^2^H KIE experiments for the arylation of benzo[*b*]thiophene, although at C3 [13]. In contrast to these pathways, the radical trap and dark experiments reported above indicate a hybrid Pd(I) radical species induced by visible light is involved in the catalytic cycle. This type of mechanism has been previously proposed for aryl and alkene alkylations [27–28], but not for direct arylation systems.

**Scheme 3:**
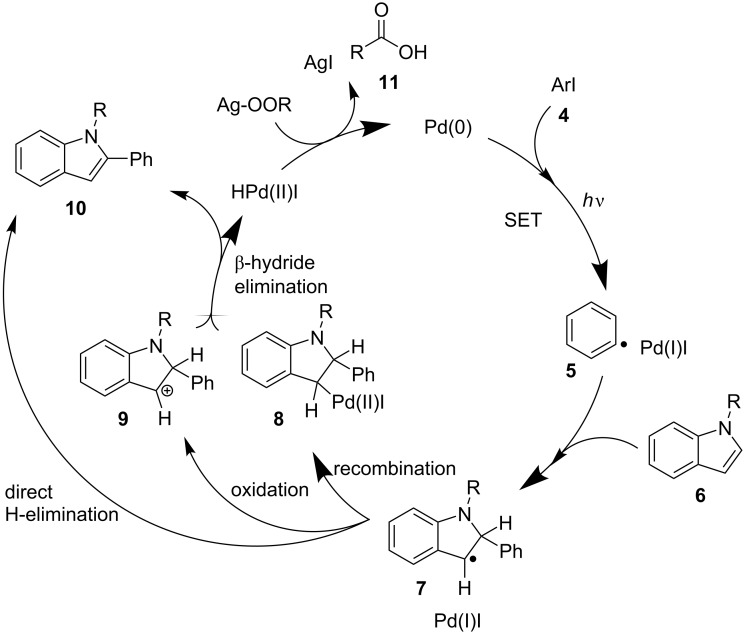
Proposed mechanism for palladium radical involved reaction between indole and iodobenzene.

A possible mechanism is outlined in Scheme 3, informed by the previous reports [27–30]. The aryl iodide **4** undergoes SET with an excited palladium(0) species to form hybrid palladium-radical intermediate **5**. This carbon-centered radical can then add to the indole. From here, three different pathways to rearomatize **7** are possible, eventually affording the arylated product **10**. Pd(0) can be regenerated by a base; in this case, the silver carboxylate. This accounts for the formation of a carboxylate radical **11**, explaining the presence of 2-nitrophenyl incorporation into the polymer chain. Additionally, this explains the formation of a phenyl radical at room temperature, and the poor control in which the nitrophenyl groups are incorporated into the chain.

There are limited examples across the aryl C–H functionalization literature indicating a transition metal-catalyzed radical process, limited largely to cobalt catalysis [31–32]. While there are several reports of palladium-catalyzed systems for room-temperature direct arylation (i.e., no directing group, transition-metal catalyzed), a radical mechanism has not been previously discussed. Some of these reports indicate a marked difference in selectivity between elevated and ambient temperatures, which we found may be due to a change in mechanism (Supporting Information File 1, Table S1, Figure S1) [11].

**Scheme 4:**
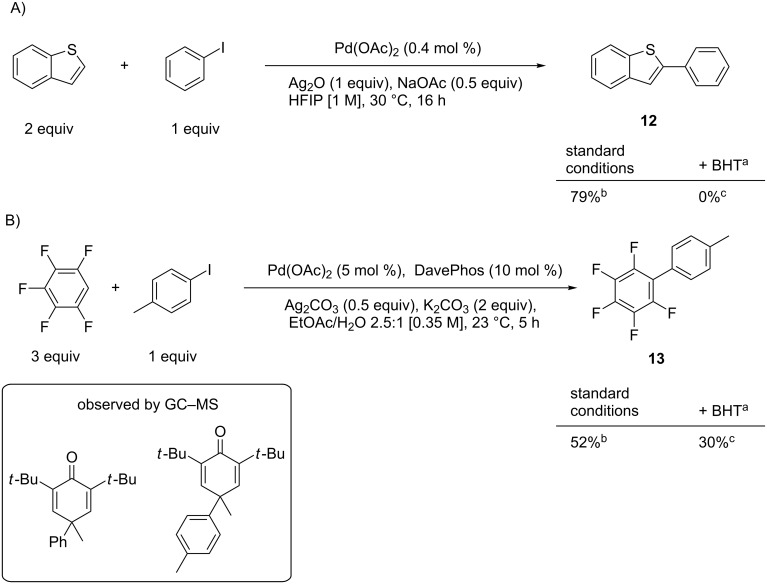
Radical trap effects on literature methods for the direct arylation at room temperature. A) From ref. [11]; B) from ref. [10]; ^a^1.1 equiv BHT added to the reported conditions; ^b^isolated yield; ^c^determined by ^1^H NMR against an internal standard.

We hypothesized that other known palladium-catalyzed room-temperature direct arylation methods could proceed using a single electron process. Two such Pd/Ag methods were investigated: a method for benzothiophene arylation [11], and a method for fluoroarene coupling [10] (Scheme 4). First the published results were replicated, then radical traps were introduced. In these cases, BHT completely eliminated the formation of product **12** and partially inhibited the conversion to product **13**. In both cases the arene–BHT adduct was observed by GC–MS as shown in the inset of Scheme 4. While more extensive studies are needed to conclusively indicate a radical mechanism, the trap experiments and room-temperature reactivity indicate that a radical-mediated mechanism similar to the indole direct arylation may be governing the observed reactivity.

## Conclusion

In conclusion, in the midst of investigating room-temperature DArP, we have discovered room-temperature palladium-catalyzed direct arylation may often be governed by radical processes. Based on the use of radical scavengers and experiments in the dark, it has become evident that a light-mediated SET is likely involved in the reported coupling of indole with iodobenzene. Moreover, other substrates such as benzo[*b*]thiophene and pentafluorobenzene appear to undergo a radical-mediated Pd/Ag room-temperature direct arylation. This is a useful insight for advancing the direct arylation knowledge base, and serves as inspiration for designing new polymerization systems that proceed under mild conditions, improving energy requirements and scalability. Work is ongoing to leverage this insight into new direct arylation methods utilizing palladium-involved radicals.

## Supporting Information

File 1 Additional condition screenings, experimental procedures, and compound characterization including ^1^H and ^13^C NMR spectra.
