# Supplementary material for: Room-temperature Pd/Ag direct arylation enabled by a radical pathway
Source: Beilstein J Org Chem. 2020 Mar 13;16:384–90. doi: 10.3762/bjoc.16.36 (PMC7082708; doi:10.3762/bjoc.16.36)
Supplement: File 1 — Additional condition screenings, experimental procedures, and compound characterization including 1H and 13C NMR spectra. [file Beilstein_J_Org_Chem-16-384-s001.pdf]

BEILSTEIN JOURNAL OF ORGANIC CHEMISTRY

Supporting Information

for

Room-temperature Pd/Ag direct arylation enabled by a radical pathway

Amy L. Mayhugh and Christine K. Luscombe

Beilstein J. Org. Chem. **2020,** *16,* 384–390. doi:10.3762/bjoc.16.36

Additional condition screenings, experimental procedures, and compound characterization including ^{1}H and ^{13}C NMR spectra

License and Terms: This is a supporting information file under the terms of the Creative Commons Attribution License (https://creativecommons.org/licenses/by/4.0). Please note that the reuse, redistribution and reproduction in particular requires that the authors and source are credited.
The license is subject to the *Beilstein Journal of Organic Chemistry* terms and conditions: (https://www.beilstein-journals.org/bjoc)

Table of Contents

High temperature conditions S2
Additional radical trap experiments S2
General information S3
Direct arylation procedures S3
Starting materials characterization S4
Products characterization S4
References S6
Polyindole characterization S7
NMR Spectra S9

High temperature conditions:

Table S1: High temperature trials

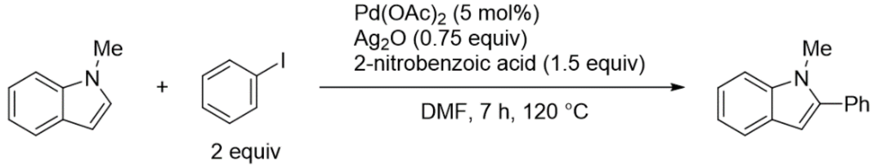

	Variation	Conversion (%)[a]	Yield (%)[a]
1	None	99	63
2	Dark	99	70
3	+ BHT[b]	47	25

[a]Determined by ^{1}H NMR using ethylene carbonate as an internal standard. [b]1.1 equiv used. Other products include 2,3-dipheny-1-methylindole.

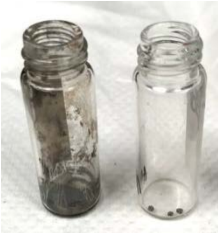

RT   120 °C

Figure S1: Silver mirroring is not observed at high temperature.

Additional radical trap experiments:

Table S2: 1-Octylindole and iodobenzene radical trap experiments.

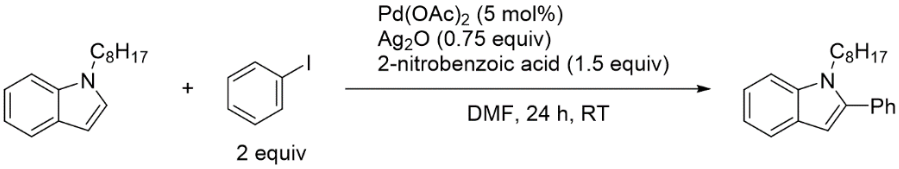

	Variation	Yield (%)[a]
1	+ TEMPO[a,b]	Trace
2	Dark	Trace
3	No $Pd(OAc)_2$	0

[a]Determined by GC–MS. [b]1.1 equiv used.

General information:

All reactions were carried out under nitrogen atmosphere using standard Schlenk techniques unless otherwise noted. NMR spectra were recorded on Bruker AV-300 and AV-500 spectrometers operating at 300 and 500 MHz, respectively. NMR chemical shifts (δ) are reported in parts per million (ppm) downfield of tetramethylsilane and are referenced relative to the residual solvent signal for ^{1}H NMR ($CDCl_3$ (7.26 ppm)) and ^{13}C NMR ($CDCl_3$ (77.16 ppm). ^{19}F NMR was referenced against hexafluoroisopropanol (-76.4 ppm). When indicated, ethylene carbonate was used as an internal standard for quantitative NMR and stored under nitrogen between uses. An Agilent 5973 Gas Chromatograph - Mass Spectrometer (EI) was used to perform GC–MS analysis. MALDI–TOF MS was collected using a Bruker AutoFlex II instrument with a terthiophene matrix. DMF and THF were dried and degassed on an Inert PureSolv solvent purification system. Deuterated solvents were stored over 4 Å molecular sieves. All other reagents and chemicals were purchased from Sigma-Aldrich or Tokyo Chemical International and used without purification. Column chromatography was performed using VWR Common Silica Gel 60 Å.

Direct arylation procedures:

Polymerizations: To a flame-dried Schlenk tube was added $Pd(OAc)_2$ (5 mol %, 0.025 mmol), Ag_2O (0.75 equiv, 0.15 mmol), and 2-nitrobenzoic acid (1.5 equiv, 0.3 mmol). Radical trapping agents were added at this time (1.1 equiv, 0.22 mmol). The flask was evacuated and backfilled with N_2 three times. DMF was added (0.5 M), followed by iodoindole (1 equiv, 0.2 mmol). The reaction was allowed to stir at room temperature (22 °C). After the required time, the reaction mixture was filtered through silica using CH_2Cl_3 to transfer and concentrated to dryness. The product was purified using Soxhlet extraction under N_2 with MeOH and collected with $CHCl_3$.

General procedure – small molecule: Based on the literature method [1]. To an oven-dried vial was added $Pd(OAc)_2$ (5 mol%, 0.025 mmol), Ag_2O (0.75 equiv, 0.15 mmol), 2-nitrobenzoic acid (1.5 equiv, 0.3 mmol), and BHT if used (1.1 equiv, 0.22 mmol). The flask was evacuated and backfilled with N_2 three times. DMF was added (0.5 M), followed by indole (1 equiv, 0.2 mmol) and the iodoarene (2 equiv, 0.4 mmol). The reaction was allowed to stir at room temperature (22 °C). After the required time, the reaction mixture was filtered through silica using CH_2Cl_2 to transfer and concentrated. Purification was performed using column chromatography (hexanes/EtOAc).

Dark trials: The general procedures were performed with the following changes: before adding reagents, the flask is wrapped in aluminum foil. After the allotted time, while still wrapped in foil the reaction mixture is quenched with 2 mL saturated EDTA solution, extracted with 5 mL CH_2Cl_2 three times, dried over $MgSO_4$, filtered, and concentrated.

Starting materials characterization:

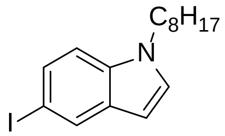

5-Iodo-1-octylindole (1): A flame-dried 3-necked round bottomed flask was charged 5-iodoindole (1 equiv, 4.1 mmol). THF was added (10 mL). The flask was cooled to −78 °C and sodium hydride (60% dispersion in mineral oil, 1.2 equiv, 4.9 mmol) was added against N_2. After stirring for one hour, iodooctane (1.5 equiv, 6.2 mmol) was added and the reaction was allowed to stir overnight at room temperature. The mixture was diluted with H_2O, and extracted 3 times with Et_2O. The combined organic layers were washed with brine, dried over $MgSO_4$, filtered, concentrated, and purified by column chromatography (hexanes with EtOAc gradient) to obtain a yellow oil (58%).
R_f = 0.3 (hexanes)
^{1}H NMR (300 MHz, $CDCl_3$) δ 7.99 (t, *J* = 1.6 Hz, 1H), 7.47 (dd, *J* = 8.7, 1.6 Hz, 1H), 7.13 (d, *J* = 8.6 Hz, 1H), 7.07 (d, *J* = 3.1 Hz, 1H), 6.43 (d, *J* = 3.1 Hz, 1H), 4.08 (t, *J* = 7.1 Hz, 2H), 1.82 (p, *J* = 7.1 Hz, 2H), 1.31 (q, *J* = 4.5, 3.8 Hz, 10H), 0.92 (td, *J* = 6.8, 1.7 Hz, 3H).
^{13}C NMR (126 MHz, $CDCl_3$) δ 135.13, 131.21, 129.89, 129.69, 129.61, 129.52, 128.64, 111.46, 100.34, 82.74, 46.58, 31.85, 30.26, 29.24, 27.02, 22.71. Spectra shown in Figure S4.
GC–MS (EI) calculated for [M]+ 355.1, found 355.1

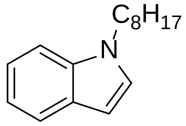

1-Octylindole: Prepared according to the literature procedure, and exhibited matching spectral data [2] (Figure S5). A clear oil was obtained in 65% yield.
R_f = 0.25 (hexanes)
^{1}H NMR (500 MHz, $CDCl_3$) δ 7.78 (d, *J* = 7.9 Hz, 1H), 7.46 (d, *J* = 8.2 Hz, 1H), 7.34 (t, *J* = 7.6 Hz, 1H), 7.28 – 7.21 (m, 1H), 7.21 – 7.17 (m, 1H), 6.62 (t, 1H), 4.19 (t, *J* = 7.2 Hz, 1H), 1.97 – 1.91 (m, 2H), 1.45 – 1.38 (m, 11H), 1.03 (t, *J* = 7.0 Hz, 2H).
^{13}C NMR (126 MHz, $CDCl_3$) δ 135.92, 128.56, 127.67, 121.21, 120.86, 109.31, 100.77, 46.30, 31.75, 30.19, 29.13, 26.96, 22.58, 14.04.
GC–MS (EI) calculated for [M]+ 229.2, found 229.3

Products Characterization:

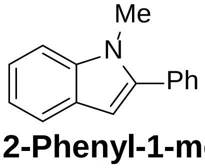

2-Phenyl-1-methylindole (3): prepared according to the literature procedure and exhibited matching spectral data [1] (Figure S6). White crystals were obtained in 85% yield.
R_f = 0.2 (98:2 hexanes:EtOAc)

^{1}H NMR (300 MHz, $CDCl_3$) δ 7.64 (d, *J* = 7.7 Hz, 1H), 7.58 – 7.32 (m, 6H), 7.15 (t, *J* = 7.4 Hz, 1H), 6.57 (s, 1H), 3.76 (s, 3H).
^{13}C NMR (126 MHz, $CDCl_3$) δ 141.57, 138.36, 132.87, 129.38, 128.48, 127.97, 127.85, 121.66, 120.47, 119.85, 109.59, 101.66, 31.15.
GC–MS (EI) calculated for [M]+ 207.1, found 207.1

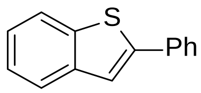

2-Phenylbenzothiophene (12): Prepared according to the literature procedure and exhibited matching spectral data [3] (Figure S7). The title compound was obtained as a white solid in 79% yield.
R_f = 0.4 (hexanes)
^{1}H NMR (300 MHz, $CDCl_3$) δ 7.84 (d, J = 7.4 Hz, 1H), 7.78 (d, J = 7.0 Hz, 1H), 7.73 (d, J = 7.2 Hz, 2H), 7.55 (s, 1H), 7.44 (t, J = 7.4 Hz, 2H), 7.34 (ddd, J = 8.6, 5.3, 1.7 Hz, 3H).
^{13}C NMR (126 MHz, $CDCl_3$) δ 140.70, 139.52, 134.31, 128.95, 128.27, 126.51, 124.51, 124.31, 123.56, 122.27, 119.45.
GC–MS (EI) calculated for [M]+ 210.1, found 210.2

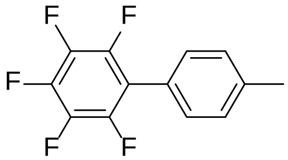

4-Methyl-2’,3’,4’,5’,6’-pentafluoro-1,1’-biphenyl (13): Prepared according to the literature procedure, and exhibited matching spectral data [4,5] (Figure S8). The title compound was obtained as a white solid in 52% yield.
R_f = 0.45 (hexanes)
^{1}H NMR (300 MHz, $CDCl_3$) δ 7.31 (s, 4H), 2.43 (s, 3H).
^{13}C NMR (excluding C_6F_5, 75 MHz, $CDCl_3$) δ 139.58, 130.15, 129.61, 123.57, 21.45.
^{19}F NMR (470 MHz, $CDCl_3$) δ -144.50 (dd, J = 26.3, 9.4 Hz), -157.25 (t, J = 22.4 Hz), -162.45 -164.41 (m).
GC–MS (EI) calculated for [M]+ 258.2, found 258.1

Radical trap adducts:

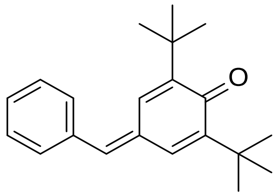

GC–MS (EI) calculated for [M]+ 294.2, found 294.3

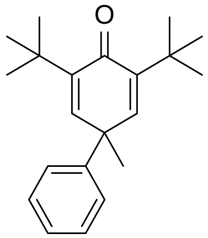

GC–MS (EI) calculated for [M]+ 296.2, found 296.3

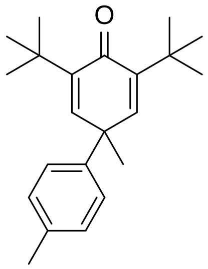

GC–MS (EI) calculated for [M+] 310.2, found 310.2

References:

(1) Lebrasseur, N.; Larrosa, I. *J. Am. Chem. Soc.* **2008,** *130* (10), 2926–2927.

(2) Sun, Q. K.; Liu, W.; Ying, S. A.; Wang, L. L.; Xue, S. F.; Yang, W. J. *RSC Adv.* **2015,** *5* (89), 73046–73050.

(3) Colletto, C.; Panigrahi, A.; Fernández-Casado, J.; Larrosa, I. *J. Am. Chem. Soc.* **2018,** *140* (30), 9638–9643.

(4) René, O.; Fagnou, K. *Org. Lett.* **2010,** *12* (9), 2116–2119.

(5) Korenaga, T.; Kosaki, T.; Fukumura, R.; Ema, T.; Sakai, T. *Org. Lett.* **2005,** *7* (22), 4915–4917.

Polyindole characterization:

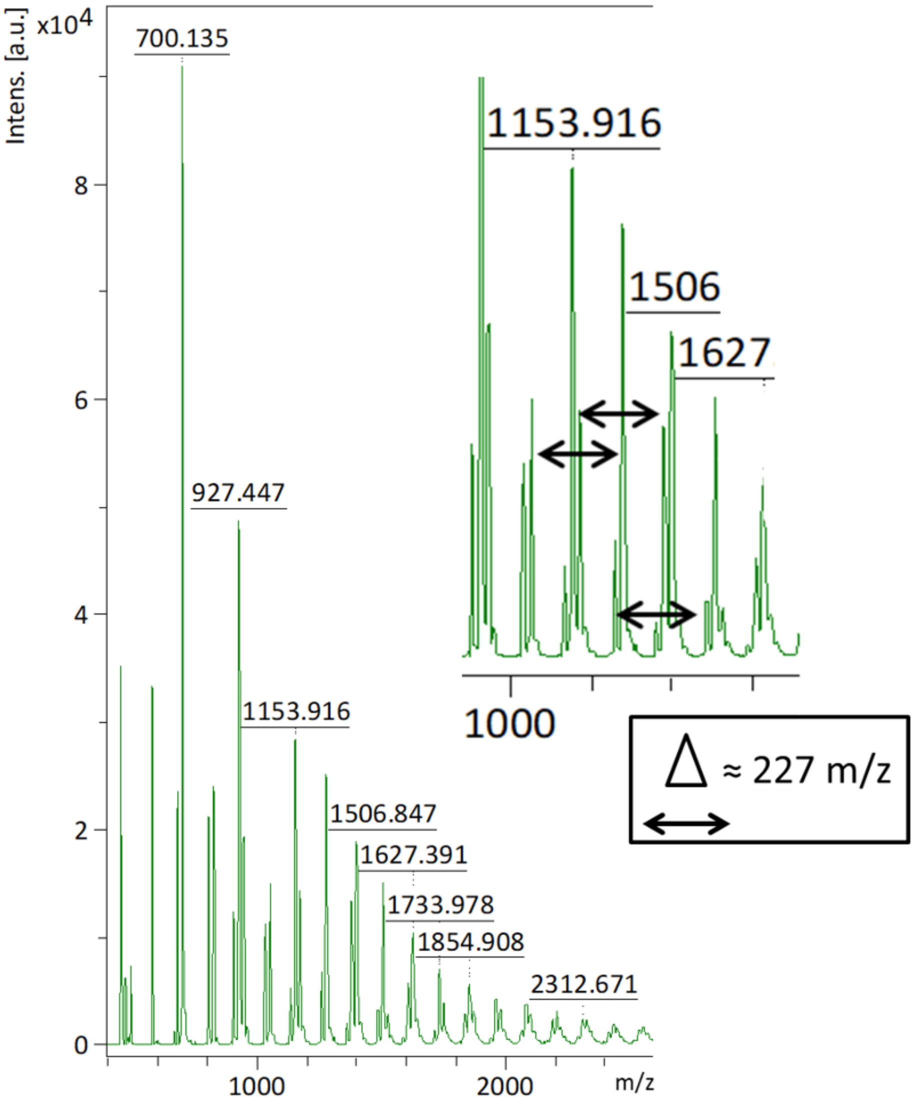

n	2 C_6H_4-NO_2 / I	C_6H_4-NO_2 / H	2 C_6H_4-NO_2
1	-	-	472
2	826	579	700
3	1053	806	927
4	1279	1033	1153
5	1506	1260	1380
6	1733	1487	1608
		1493	

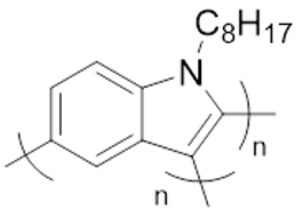

Figure S2: MALDI–TOF MS for branched polyindole, with key peaks summarized in the table based on end group identity.

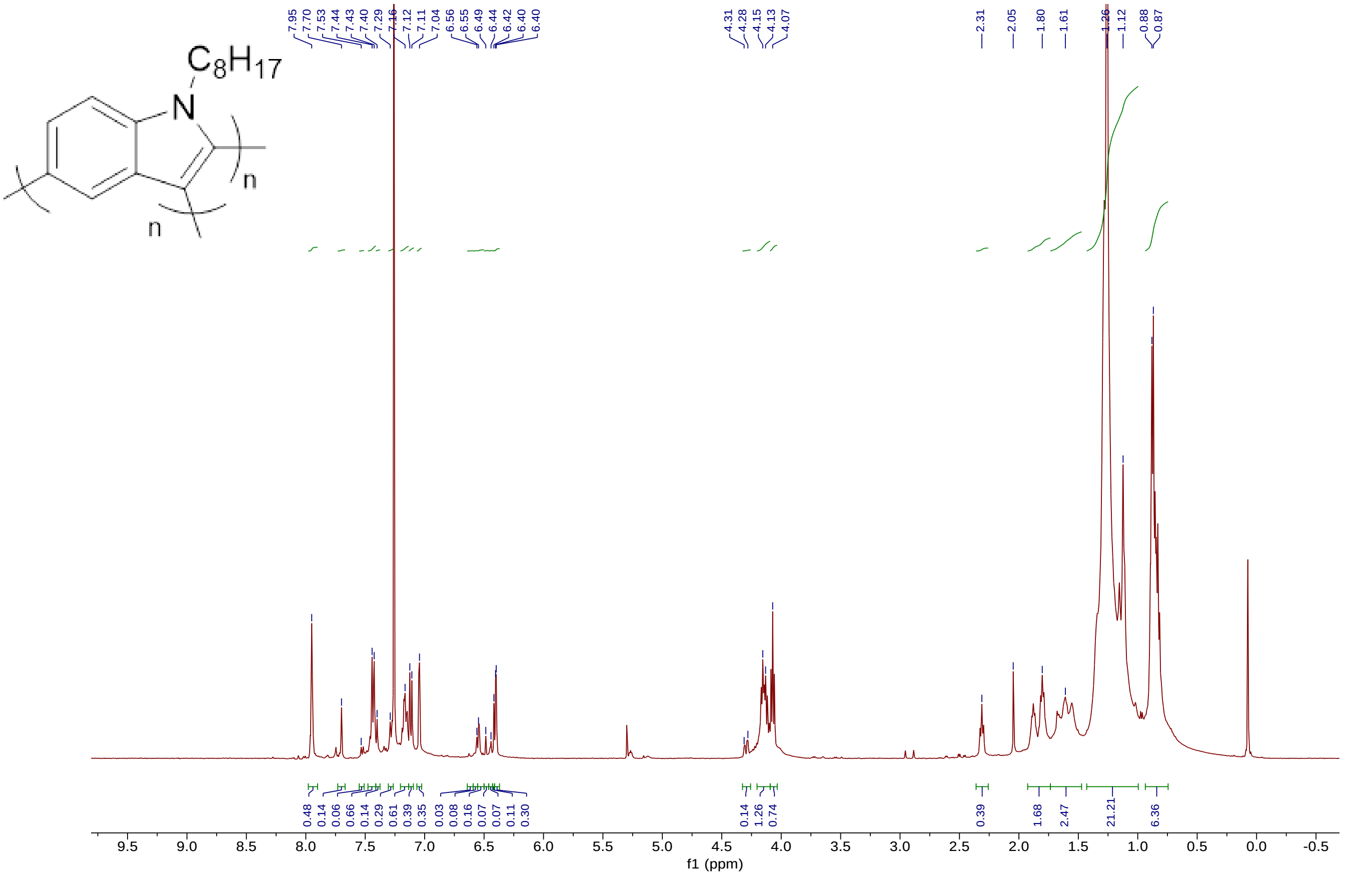

Figure S3: ^{1}H NMR of polyindole .

NMR Spectra

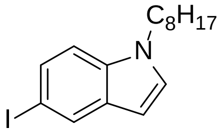

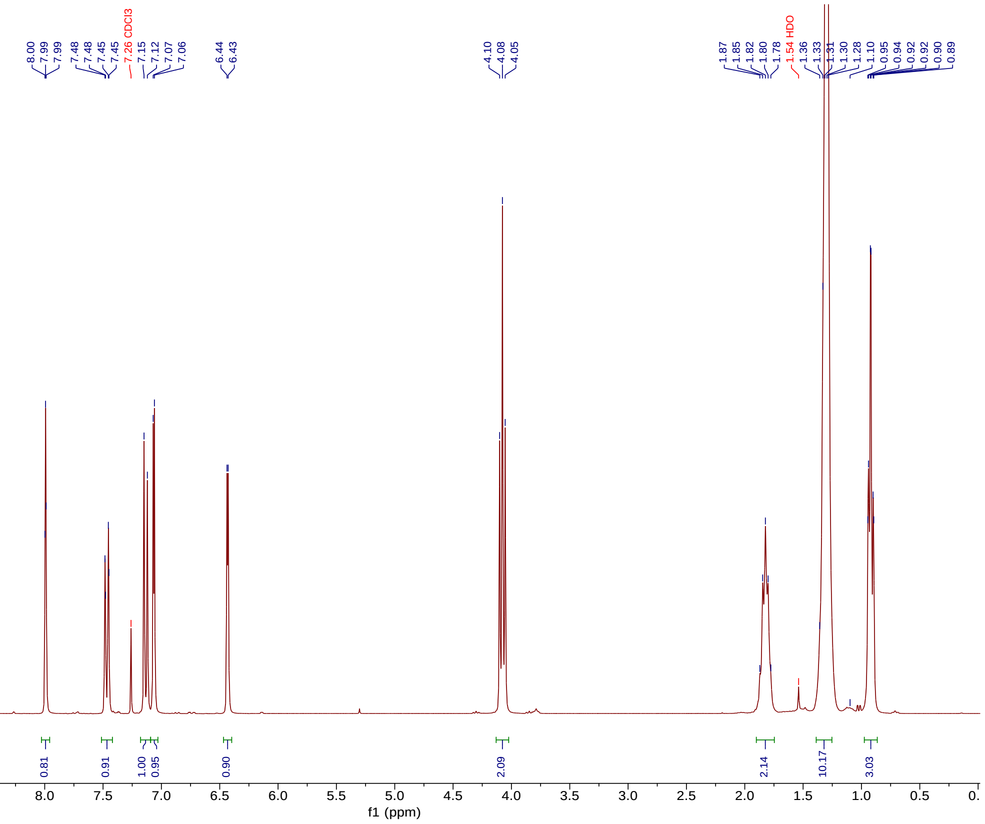

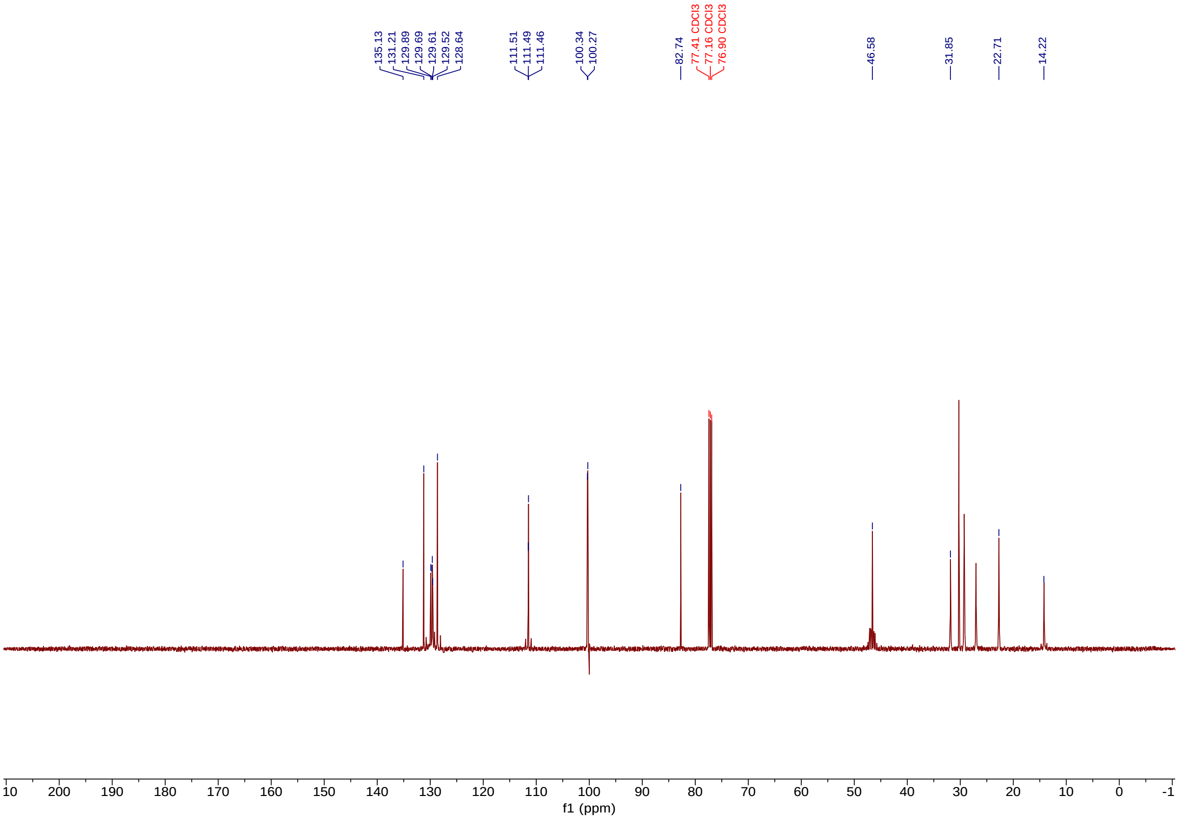

Figure S4: ^{1}H NMR (top) and ^{13}C NMR (bottom) of **1**.

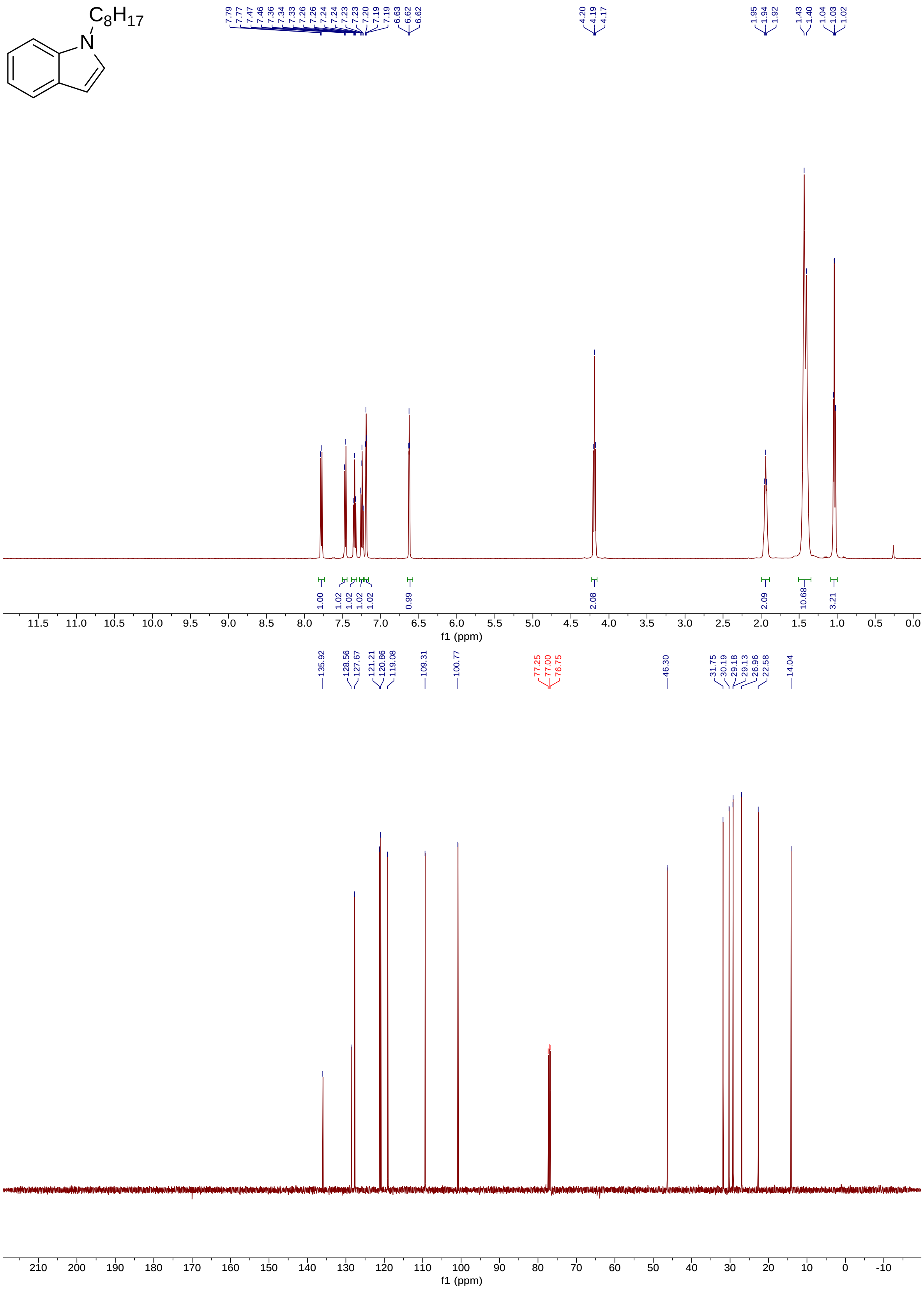

Figure S5: ^{1}H NMR (top) and ^{13}C NMR (bottom) of 1-octylindole.

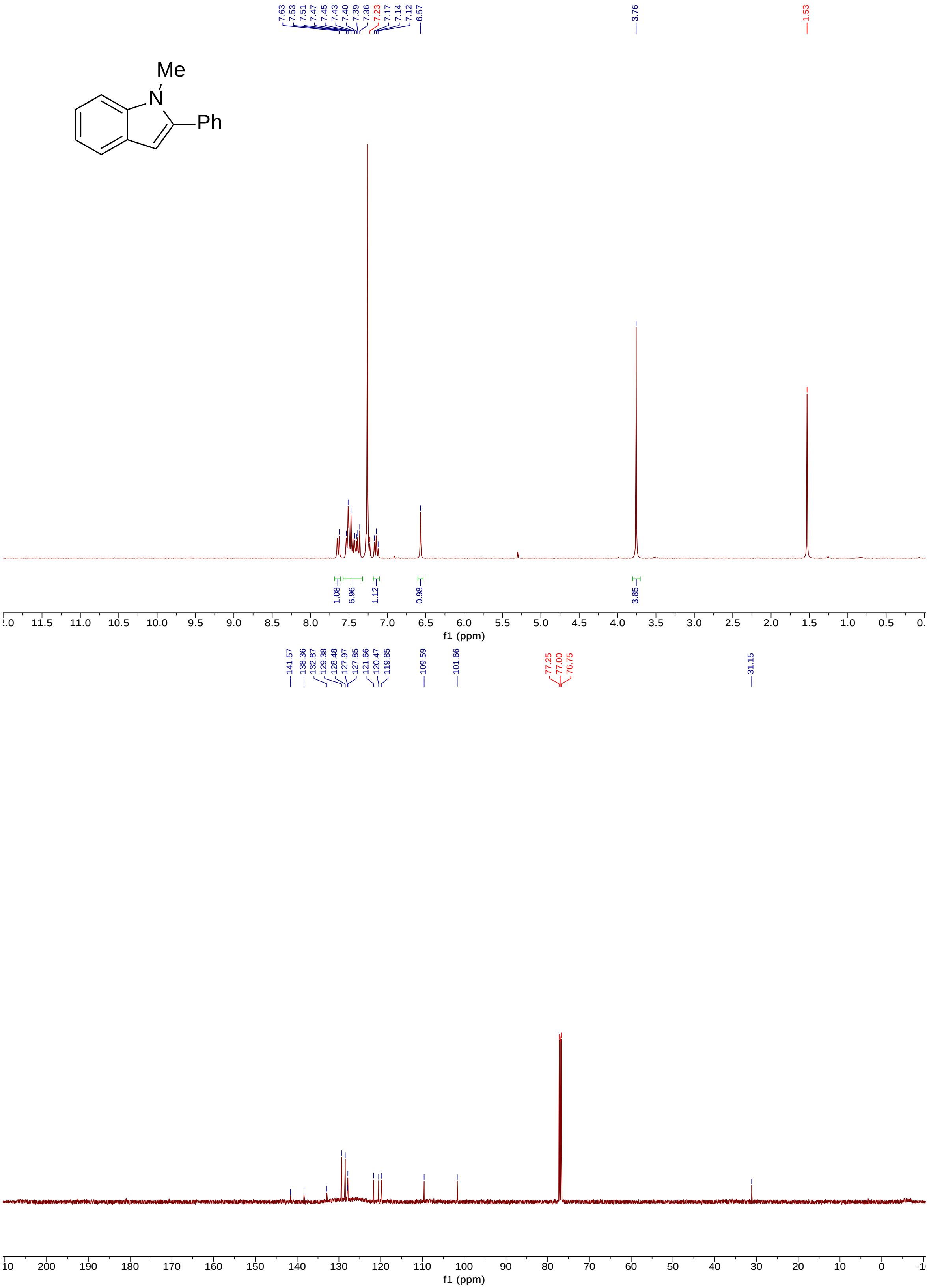

Figure S6: ^{1}H NMR (top) and ^{13}C NMR (bottom) of **3**.

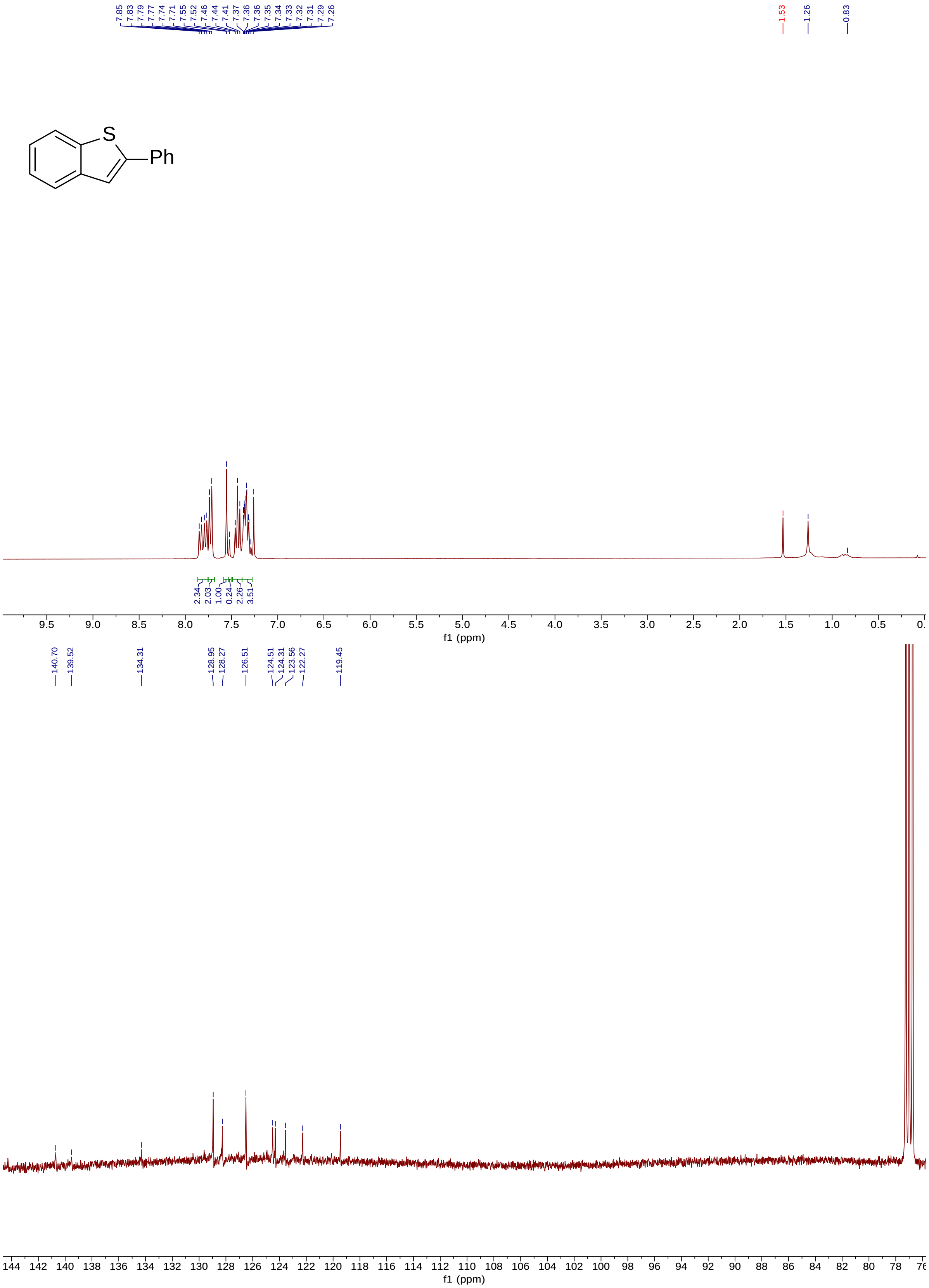

Figure S7: ^{1}H NMR (top) and ^{13}C NMR (bottom) of **12**.

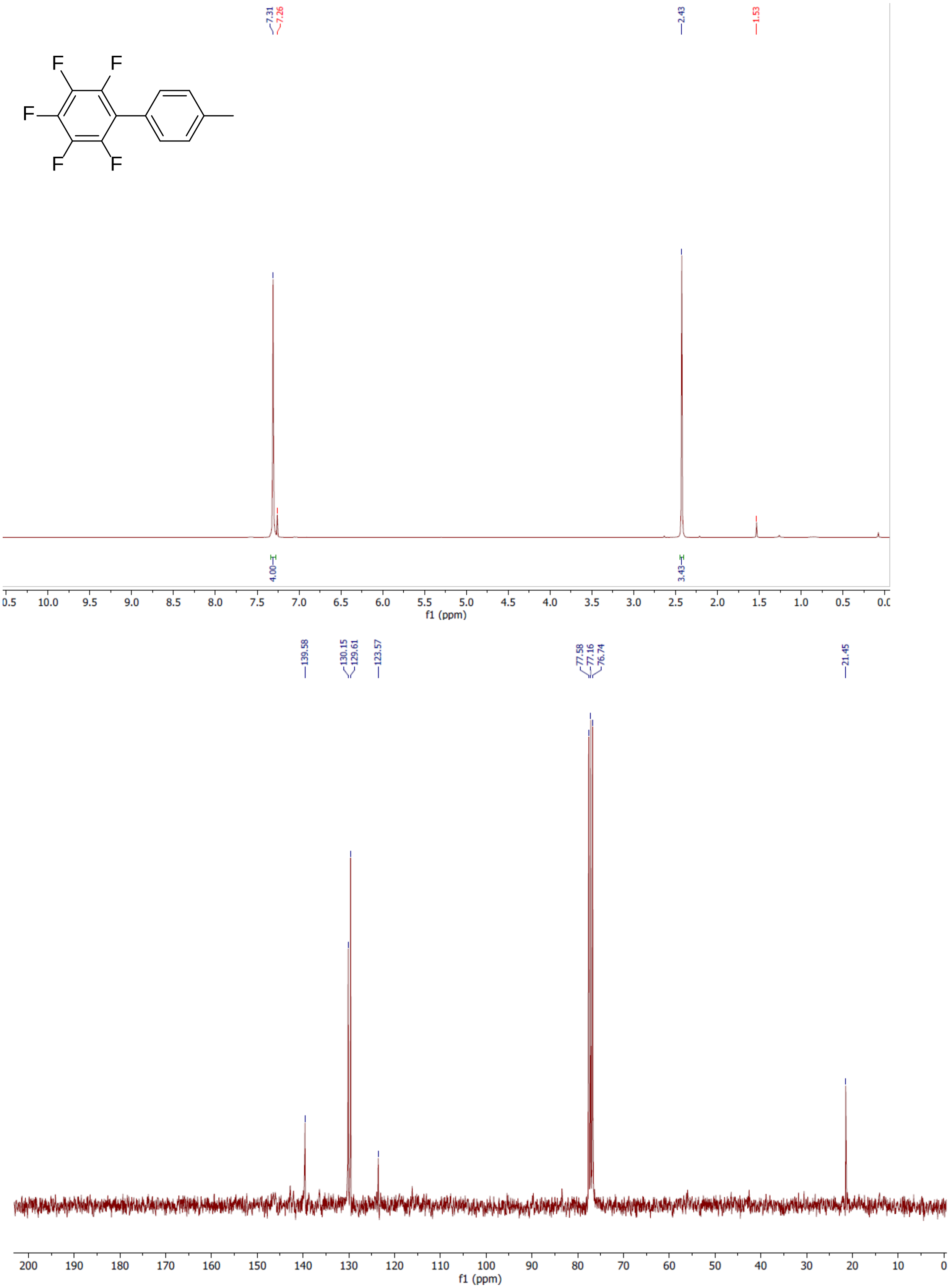

Figure S8: ^{1}H NMR (top) and ^{13}C NMR (bottom, excluding C_6F_5) of **13**.

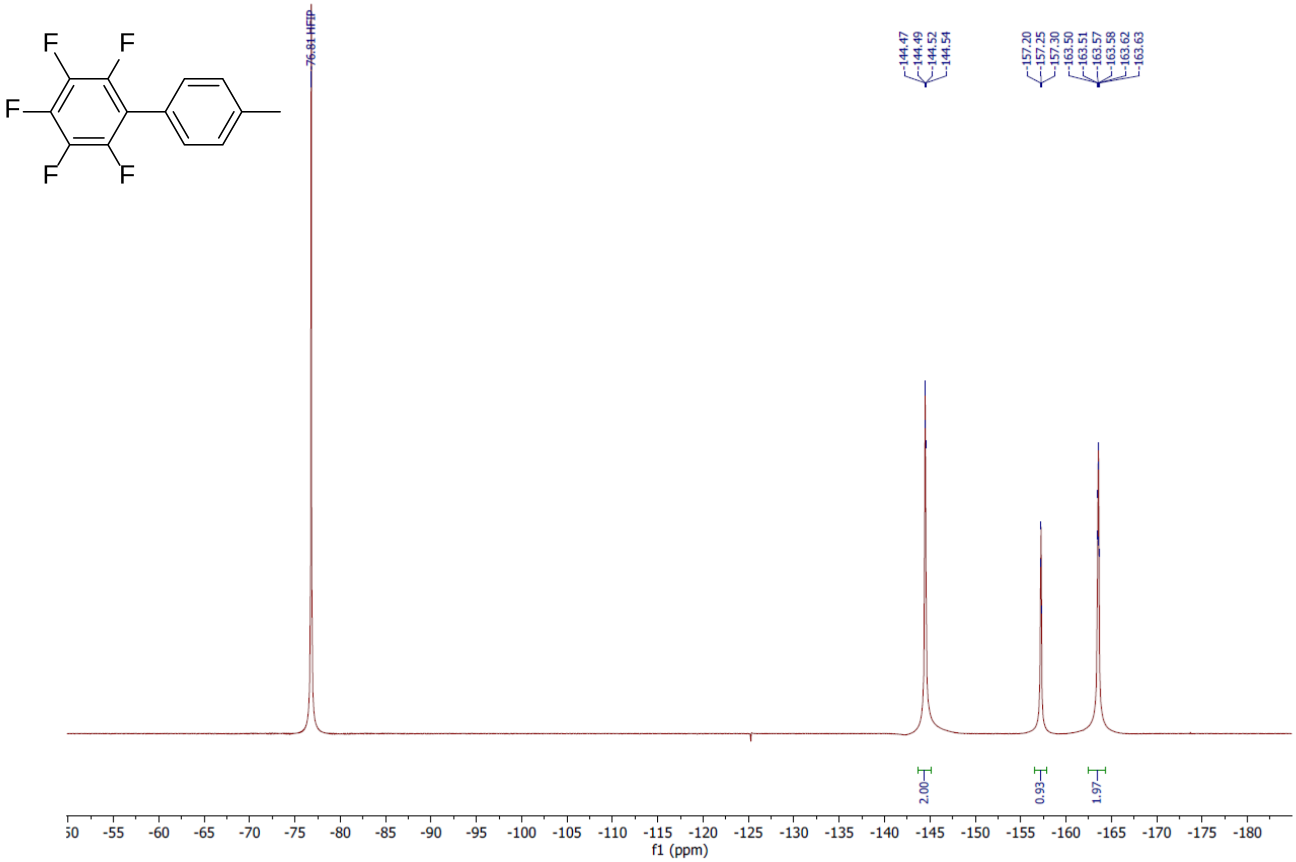

Figure S9: ^{19}F NMR of **13**.
